# Self-Healing of Cracks in Cementitious Materials as a Method of Improving the Durability of Pre-Stressed Concrete Railway Sleepers

**DOI:** 10.3390/ma17030760

**Published:** 2024-02-05

**Authors:** Marta Dudek, Teresa Stryszewska

**Affiliations:** Chair of Building Materials Engineering, Faculty of Civil Engineering, Cracow University of Technology, 24 Warszawska St., 31-155 Cracow, Poland; teresa.stryszewska@pk.edu.pl

**Keywords:** durability, self-healing, concrete, cementitious materials, tubes, polyurethane, cracks, cement beams, concrete beams, railway sleepers

## Abstract

The article presents research results regarding the possibility of modifying pre-stressed concrete railway sleepers to improve their durability. The cracks that appear in these elements are one of the reasons for shortening the period of safe use. They do not have a significant impact on the load-bearing capacity of these elements, but on their durability. The resulting scratches become an easy way for the external environment to migrate inside the element, including the reinforcement area. Despite efforts to eliminate the possibility of cracking, this phenomenon still occurs in railway sleepers. In order to reduce the negative effects of cracking the cement matrix, a technology for modifying a prefabricated concrete element with resin-filled tubes towards its autonomous self-healing was developed and tested. The tests were divided into three stages, including laboratory tests carried out on cement mortar beams, semi-technical tests carried out on reinforced concrete beams, and industrial tests carried out on pre-stressed concrete and prefabricated railway sleepers. All research conducted on a laboratory and semi-technical scale, preceding the target stage, was intended to ultimately enable the development of tube application technology on an industrial scale while verifying the effectiveness of self-healing at the laboratory level. The use of self-healing cementitious materials potentially reduces the negative effects of cracking railway sleepers, as shown by observations conducted during the research.

## 1. Introduction

### 1.1. Factors Influencing the Durability of Pre-Stressed Concrete Railway Sleepers

Railway sleepers are one of the elements of the railway surface. A typical railway surface is composed of ballast, sleepers with fastening systems and rails. The purpose of the sleepers is to maintain the appropriate rail spacing and transfer the loads from the rails to the ballast. Modern railway roads are constructed mainly using pre-stressed concrete sleepers, the minimum durability of which is estimated at approximately 40 years. Prestressing is the intentional introduction of stresses into an element that will counteract the load stresses arising as a result of exploitation.

The location of the crack in the concrete sleeper depends on the nature of the load. During the passage of the rolling stock, the lower parts of the under-rail zone and the upper part of the central zone are stretched. As a result of cyclic loading and unloading of the railway track during the passage of rolling stock, cracks are formed in the part under the rails from the lower surface of the sleeper. For the same reason, cracks may also appear in the central part from the upper side. In the central zone, on the lower surface of the sleeper, cracks may appear due to assembly work [1]. While the formation of a crack itself is not dangerous from the point of view of load-bearing capacity, it is dangerous from the point of view of the durability of this element. Therefore, it is right and legitimate to take action aimed at limiting the effects of cracking pre-stressed concrete railway sleepers in terms of maintaining their durability.

In addition to mechanical impacts, prestressed concrete sleepers are also exposed to physical and chemical impacts from the external environment. Therefore, the standardized scope of sleeper testing concerns both mechanical and durability aspects. A characteristic parameter responsible for the durability of sleepers is their crack resistance. In many cases, under the influence of an external load, a slight scratch on the sleeper disqualifies it. The direction of application proposed in this work is consistent with the concept of filling the crack that has occurred and, consequently, protecting the prestressing reinforcement against access to the external environment through the damaged cover. The choice of this element was not accidental, because in the case of these elements, there is a natural need to minimize the effects of cracks. After the first crack appears, the sleeper still meets the load-bearing criterion and is able to transfer loads, but it becomes more susceptible to environmental influences, and sealing the crack can significantly reduce this. Healing the crack will have a positive impact on the durability aspects, not the mechanical ones.

### 1.2. Methods Used in the Self-Healing of Cementitious Materials

Recent years indicate a great interest in self-healing materials, which transfer into their continuous development and multifaceted research in this field. Self-healing means the processes taking place in the material that consequently lead to its self-repair, initiated by the components of this material. This happens without any external action. In cementitious materials characterized by brittle destruction and the ability to crack, the self-healing process consists of filling the cracks [2,3]. The processes that take place during this time can be divided into two groups. The first one concerns the mechanisms that are natural for the cementitious material and do not require human interference in the mix design process, that is, the so-called autogenic mechanisms. In this case, the material uses only its natural properties resulting from its composition. Modifications are not made intentionally. Autonomous mechanisms constitute the second group. This is where the human factor comes in at the material design stage, consisting of the appropriate selection of its composition or the introduction of a specific modification aimed at obtaining self-healing properties. The purpose of these mechanisms is to fill the resulting crack and thus restore the original properties of the material to the greatest extent possible [3]. Autonomic healing involves the use of mineral additives, which are the most basic self-healing mechanism in this group, as well as the addition of bacteria or carriers in the form of capsules or tubes filled with a healing agent, which are more technologically advanced methods of self-healing [4,5,6]. The addition of mineral additives in its mechanism is based on the hydration of ingredients introduced intentionally into the mixture. This process occurs as a result of the contact of additives with water after the crack occurs. The resulting products build up in the crack, sealing it. This includes the addition of fly ashes, silica fume, blast furnace slag and many others [7,8,9,10,11]. Another method is the addition of bacteria. They can be dispersed in a matrix or closed in a carrier in the form of a capsule, tube or lightweight aggregate. Bacteria are required to be able to survive in an alkaline environment. The mechanism of this method is the activation of dormant bacteria as a result of crack formation and contact with water. During the metabolism, calcite is secreted, which fills the cracks in the cementitious material [12,13,14,15,16]. The use of capsules or tubes is a method of enclosing a healing agent in a selected carrier. When the matrix is scratched, the carrier also breaks, releasing the substance contained in it. This is a more technologically demanding process. The capsules used in cementitious materials are micro-sized and dispersed in the matrix. A polymer, bacteria or mineral additive can be enclosed inside [17,18,19,20,21,22]. The same is in case of tubes. The main differences concern the shape of the carrier and its dimensions. This carrier is usually placed in the appropriate places of the structure. A polymer is usually encapsulated inside such a carrier. Although the first reports describing the autonomous self-healing process of cementitious materials with the use of tubes date from the 1990s, this method has not yet come into everyday use in structures made of cementitious materials. It was Carolyn Dry [23,24,25] about 30 years ago who proposed a solution consisting of enclosing a healing agent (in the form of cyanoacrylate) in glass fibres. This agent needs air to initiate the self-healing process. In this way, she modified the cement beams and performed mechanical tests. The results obtained by her showed the possibility of carrying a greater load by most of the modified samples after the self-healing process, compared to the values obtained originally.

This method has developed rapidly over the years, both in terms of the selection of the carrier and the healing agent. Typically, the use of a one-component agent is preferred due to the elimination of the self-mixing of ingredients in multi-component substances [26,27]. The biggest disadvantage of one-component agents that bind in the presence of air is the possibility of starting the reaction inside the tube when air bubbles are trapped in it. However, in the case of agents that bind in contact with moisture, the presence of water or moisture present in the air or in the material is necessary.

### 1.3. Purpose and Scope of Research

The aim of the research presented in this article is to check the possibility of extending the durability of pre-stressed concrete railway sleepers by filling cracks that appear in the element as a result of exploitation. For this purpose, polymer-filled tubes were introduced into the sleepers to provide self-healing potential. Filling the cracks leads to closing the migration path of the external environment into the material, thus cutting it off from harmful environmental influences that may lead to corrosion of the reinforcement.

**Figure 1:**
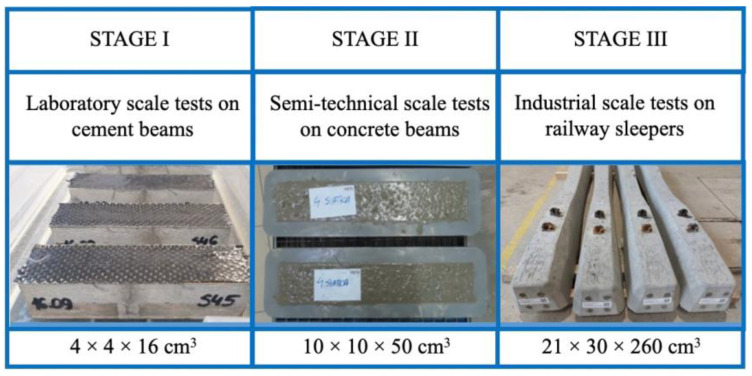
Research scheme.

The second aspect discussed is the technology of the modification itself. Research into the possibility of self-healing cement materials using various methods has been conducted for about 30 years. Despite this, many questions remain about effectiveness, research methodology and practical application. Transferring the modification of cementitious material from the laboratory scale to the industrial scale is not a simple task and is rarely discussed in the literature. Therefore, most of the research conducted remains on a laboratory scale. The authors of this publication made an attempt to transfer the method of modifying self-healing cementitious materials from the laboratory scale to the industrial scale by carrying out the following stages of work, that is, tests on the laboratory, semi-technical and industrial scales (Figure 1).

Laboratory-scale tests were carried out on cement beams with dimensions of 4 × 4 × 16 cm^3^. On a semi-technical scale, the tests were carried out on concrete beams (10 × 10 × 50 cm^3^) with two steel rods. The target application was made on prestressed railway sleepers with approximate dimensions of 21 × 30 × 260 cm^3^.

The laboratory and semi-technical tests preceding the last stage were aimed at developing a method of modifying pre-stressed concrete railway sleepers in order to increase their durability. Healing the crack will affect durability, not mechanical aspects.

## 2. Materials and Methods

### 2.1. Materials

#### 2.1.1. Glass Tubes

**Figure 2:**
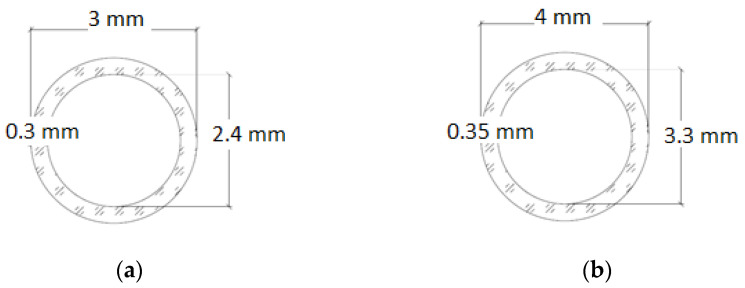
(**a**) Dimensions of S3 tube and (**b**) dimensions of S4 tube.

The basic criterion that the carrier must fulfil is the ability to survive during concreting and vibration, as well as the ability to break brittlely when the matrix is cracked, so that the healing agent can be released. Two types of borosilicate glass tubes with different diameters and wall thicknesses were selected for the tests (Figure 2). Both types of tubes have the same length of 100 mm.

The S3 tubes allow the application of 0.45 cm^3^ of the healing agent, while the S4 tubes allow 0.85 cm^3^, which is almost twice as much. The tests carried out on the laboratory and semi-technical scale made it possible to select one diameter that was ultimately used in the structural element.

#### 2.1.2. Healing Agent

In the case of a healing agent, the selection criterion concerns the possibility of enclosing it in the carrier, leaking away when the matrix is cracked, and filling the resulting crack. One-component polyurethane (PU) was used as a healing agent, which, in contact with water/moisture undergoes a foaming process, increasing its volume. In its liquid state, it has a low viscosity of 255 mPas. Depending on the access of the water medium, PU increases its volume up to 25 times. Contact with water/moisture is necessary for the foaming process. At the end of this process, it takes the form of foam with a sealed surface. PU is a material with good adhesion to concrete (0.6 N mm^−2^). It is dedicated to masonry and concrete structures as a sealing material.

**Figure 3:**
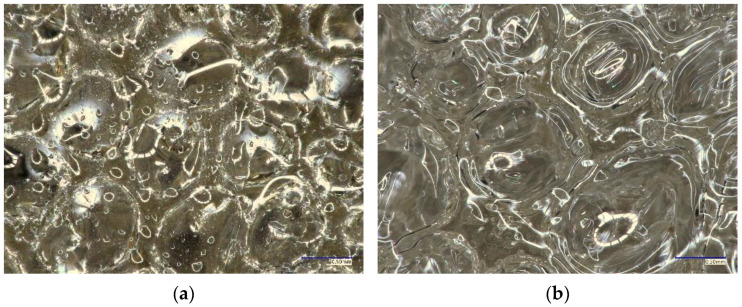
Pictures of PU taken under a digital microscope, magnification 100×: (**a**) PU0 surface and (**b**) PU5 surface.

Due to the fact that the cementitious material is a material that, depending on the exploitation conditions, can be characterized by different moisture levels, and taking into account the ability of PU to foam in contact with moisture/water, the effect of the amount of water on the PU microstructure was analysed. For this purpose, PU with different percentages of water (0%, 1%, 2%, 3%, 4%, and 5%) in relation to its weight were subjected to digital microscope observations (Table 1). The obtained results of the 0% and 5% cases are shown in the pictures below (Figure 3).

The structure of the foamed PU changes with an increase in moisture content. At trace moisture, small PU foaming was observed as well as numerous drops of liquid PU (Figure 3a) enclosed in the pores of the material. Drops of liquid PU act as a repair buffer when the element is cracked again in the same place, which means that when the crack is re-opened, it will foam and expand. The amount of non-foamed material in the liquid state (visible as drops closed in the pores) significantly decreases and even disappears with the increasing addition of water. On the other hand, the more moisture there is, the greater the expansion of PU and thus its porosity (Figure 3b).

**Figure 4:**
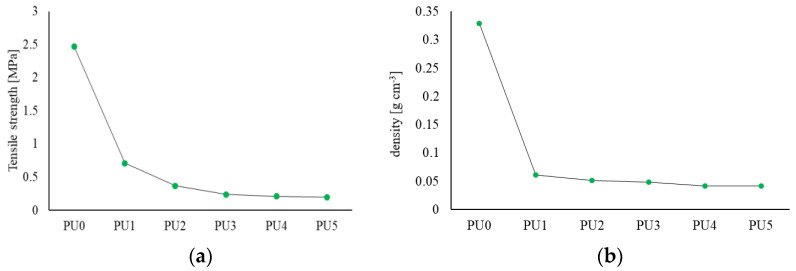
(**a**) Graph showing the average tensile strength value for polyurethane with the different percentages of addition of water; (**b**) Graph showing the density of polyurethane with the different percentages of addition of water.

As part of the research, the density and tensile strength of polyurethane samples were also tested in order to determine their mechanical properties. The tests were carried out for PU with different amounts of water, according to Table 1. From each material, 15 samples were prepared in the form of strips 5 cm long, 0.5 cm wide and 0.3 cm thick. Strength tests were performed using a tensile-stage DEBEN Microtest 200 N (Deben UK Ltd., Bury Saint Edmunds, UK) mounted in the chamber of a scanning microscope SEM. The results of the density and tensile tests are presented in the graphs (Figure 4).

Based on the research, it was observed that as the water content increases, both the density and strength of the polyurethane significantly decrease. Comparing PU0 and PU3, the difference is tenfold, while between PU0 and PU5, the difference is twelvefold. Both density and strength stabilize above 3% water addition.

#### 2.1.3. Cement Mortar

Tests on a laboratory scale were carried out on beams with dimensions of 4 × 4 × 16 cm^3^, made of a standard cement mortar with the addition of a superplasticizer. For this purpose, reference samples and samples modified with PU-filled tubes were made. The tubes were placed at a height of approximately 5 mm from the bottom surface of the beam. Two sizes of carrier, S3 and S4, were used, filled with one-component polyurethane.

#### 2.1.4. Concrete

Both for tests on a semi-technical and industrial scale, the samples were made in the manufacturer’s laboratory of railway sleepers using a concrete mix taken directly from production. Concrete with a strength class of C70/85 was used (the strength class was determined according to PN EN 206+A1 2016-12).

**Figure 5:**
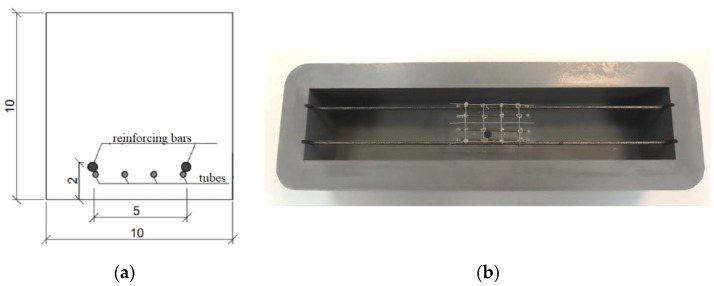
Arrangement of reinforcing bars and four tubes in the beam cross-section: (**a**) scheme presented in centimeters (**b**) photo of the prepared cast.

Within the tests on a semi-technical scale, 6 concrete beams with dimensions of 10 × 10 × 500 cm^3^ and two steel bars with a diameter of 5 mm were made. Tubes filled with polyurethane were placed in the central zone of the samples (Figure 5).

Within the six beams made, four S3 tubes were placed in three of them, and four S4 tubes in the next three.

As part of the application tests, 6 PS-94/SB concrete sleepers were made in an industrial laboratory, modifying them with PU-filled tubes in selected cross-sections. As reinforcement, steel wires Ø7 mm Y1670C were used to prestress the structure with a characteristic tensile strength of 1670 MPa.

**Figure 6:**
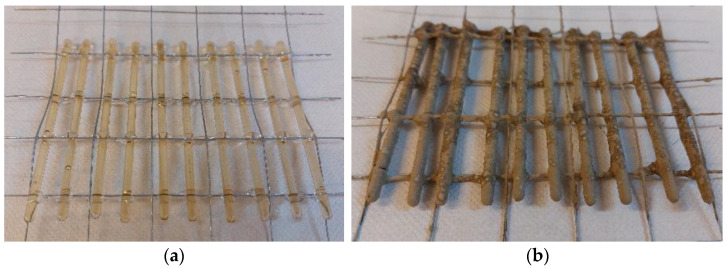
S4 tubes filled with polyurethane, placed on a steel mesh, prepared for installation in concrete sleepers: (**a**) before covering with mortar and (**b**) after applying a layer of mortar.

Two potentially most strenuous cross-sections of sleepers were selected for modification, that is, the middle part and the part under the rail. S4 tubes were used. There were 10 carriers for one selected cross-section in the railway sleeper. They were attached to the reinforcement using a steel mesh with a 0.7 mm diameter wire and square meshes of 25 × 25 mm^2^. The method of placing the PU-filled tubes in the cross-section of the sleeper was determined based on the tests carried out on concrete beams. All carriers were filled with polyurethane and attached to the meshes to prevent their displacement (Figure 6). As the concrete mix in its form is intensively vibrated during the production process of the sleepers, all the tubes and the mesh were covered with a thin layer of mortar to prevent damage to the glass carrier during the concreting of the sleepers (Figure 6).

**Figure 7:**
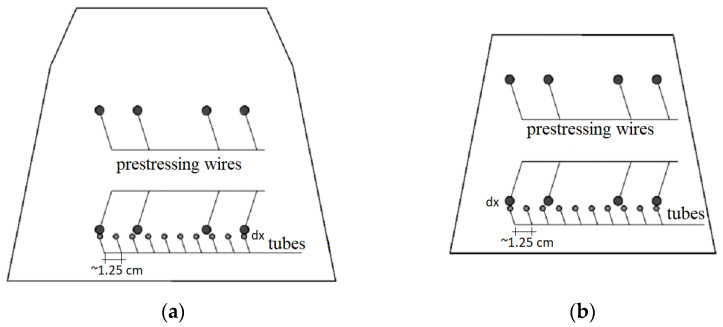
Arrangement of S4 tubes in the railway sleeper: (**a**) in the rail part and (**b**) in the middle part.

Then, the carriers prepared in this way were attached to the reinforcement in the sleeper in the parts vulnerable to cracking, that is, in the middle and the rail parts. Since it was assumed that the tests would be carried out in the rail part and in the middle part in the normal position, the tubes were placed only in the lower part of the sleepers (Figure 7).

**Figure 8:**
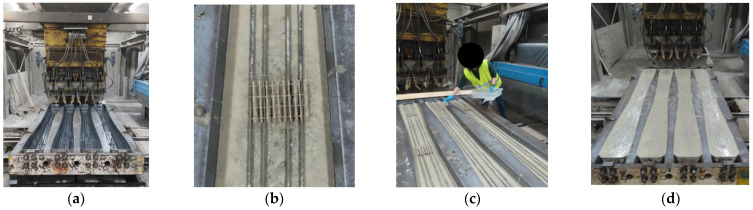
Stages of production of railway sleepers modified with tubes filled with PU: (**a**) empty casts, (**b**) arrangement of tubes attached to steel meshes, (**c**) assembly of tubes, and (**d**) moulds filled with concrete mix.

Concrete sleepers modified with tubes filled with a healing agent were made in the factory of prestressed concrete sleepers. After placing and installing the prestressing reinforcement, the casts were gradually filled with concrete mix and intensively vibrated. After filling the mould with the concrete mix to the level of the lower reinforcement, the concreting process was stopped in order to apply PU-filled tubes. Properly prepared sets of tubes were placed in selected cross-sections and attached to the prestressing wires. Then concreting was continued (Figure 8).

After casting, the sleepers were covered with foil and left in the tunnel for setting and curing. Subsequently, the compression process was carried out by releasing the tension. The last stage of the production process was demoulding the sleepers, quality control and transport to the storage yard.

### 2.2. Methods

Depending on the stage of research (on a laboratory, semi-technical and industrial scale), the methods used to assess the effectiveness of self-healing of cementitious materials by using modifications with polymer-filled tubes were different.

#### 2.2.1. Test Methods for Mortars on a Laboratory Scale

##### Water Permeability Test

For the water permeability test, 45 samples of cement mortar were prepared. Of these, 15 reference samples and 30 self-healing samples were made, of which 15 were in S3 tubes and 15 were in S4 tubes. The water permeability test was carried out based on the procedure developed under COST Action 15202 [28]. After 28 days of casting, the samples were cracked in a 3-point bending test. The cracks were limited to the set width using special holders. The average crack width for the reference samples was 298 µm. Accordingly, for the samples with S3 tubes, this value was 293 µm, and for the samples with S4 tubes, it was 295 µm. During the crack width limiting, a polymer leak could be observed. Most often, this phenomenon was observed in the beams with S4 tubes. After two days, the samples were placed on the water permeability testing stand. The prepared cast-in hole in the sample was connected to the water reservoir. The side walls where the crack runs were sealed with aluminium tape. A water flow test was then performed for each sample. The flow was measured automatically using a program implemented in Excel that reads the mass of water that leaked into a container placed on the scale under the sample. Based on the measurements, the flow rate in g min^−1^ units was calculated for reference and modified beams, and then the self-healing efficiency of the samples was calculated. This parameter was expressed as a percentage using the sealing efficiency factor (SE) [28] based on the formula below (Formula (1)). Averaged results for individual samples were used in the calculations.

(1) $$SE=\frac{q_{REF}-q_{S}}{q_{REF}}\cdot 100\%\;$$

where SE in the formula means the sealing efficiency factor (SE_S3_, SE_S4_), q_REF_ means the average value of water flow in g min^−1^ for reference samples and q_S_ means the average value of water flow in g min^−1^ for samples with tubes (q_s3_, q_s4_).

##### Water Absorption Test

For the water absorption test, 60 cement mortar samples were prepared. Of these, 15 samples are reference beams that have not been cracked (REF), and another 15 samples are reference beams that have been cracked (ZREF). The remaining 30 are cracked samples with tubes, including 15 beams with S3 tubes and 15 with S4 tubes. The samples were prepared analogously to the water permeability test, but without the flow hole. The test was based on the procedure developed by COST Action 15202 [28]. After 28 days of casting, the samples were cracked in the 3-point bending test. Immediately after breaking, the width of the crack was limited to about 300 µm with steel holders.

**Figure 9:**
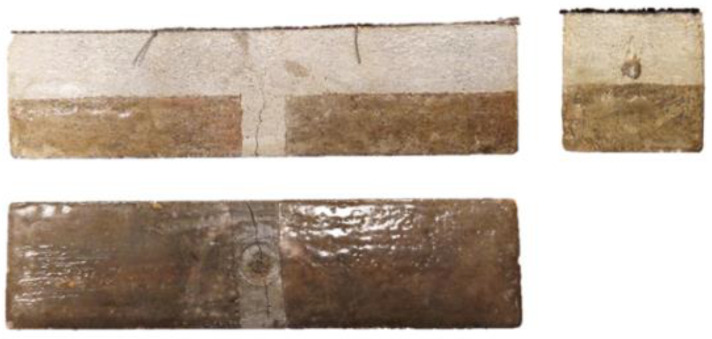
Protection of the samples with an epoxy layer.

The average crack width obtained for the reference samples (ZREF) was 307 µm. Accordingly, for the beams with S3 tubes, this value was 294 µm, and for the samples with S4 tubes, it was 295 µm. When limiting the width of the crack, a polymer leak could be observed. Most often, this phenomenon was observed in the beams with S4 tubes, similar to the samples used for the water permeability test. After limiting the crack width with steel holders, the samples were stored in laboratory conditions for 2 days. After this time, the beams were placed in a dryer at a temperature of 40 °C for about 14 days to achieve a constant weight (the difference between two consecutive measurements at a 2 h interval was not greater than 0.2%). The samples were then again stored in laboratory conditions for 24 h. Before the absorption test, part of the surface of the samples was covered with an epoxy coating (Figure 9).

After proper preparation of the samples, the water absorption was measured during 24 h, at specific time intervals (10 min, 20 min, 30 min, 1 h, 1 h 30, 2 h, 3 h, 4 h, 6 h, 8 h, and 24 h), each time recording the weight of the beams with an accuracy of 0.01 g.

To determine the percentage self-healing efficiency of the beams, the sealing efficiency factor (SE) [28] was calculated based on the following formula (Formula (2)).

(2) $$SE=\frac{{SC}_{ZREF}-{SC}_{S}}{{SC}_{ZREF}-{SC}_{REF}}\cdot 100\%$$

where SE in the formula means sealing efficiency factor (SE_S3_ and SE_S4_), SC_REF_ means the average value of the absorption coefficient for non-cracked reference samples, SC_ZREF_ means the average value of the absorption coefficient for cracked reference samples and SC_S_ means the average value of the absorption coefficient for samples with tubes (SC_S3_ and SC_S4_).

##### Macroscopic Observations

Observations were made visually, assessing how the tubes remained in the matrix in a given location and how they survived the forming and vibration processes. The ability of polyurethane to pour out of the tube and fill the crack, as well as cover the fracture surface, was also assessed.

##### Observations in a Scanning Microscope

Microscopic observations were carried out in a Zeiss EVO-MA 10 scanning electron microscope (SEM) (Carl Zeiss NTS Ltd., Oberkochen, Germany) equipped with SE, VPSE and BSD detectors and a Brucker XFLASH 6/30 EDS detector. Mortars modified with PU-filled tubes were tested. SEM observations were performed using a variable vacuum (range 80–120 Pa) and a BSD detector with EDS chemical analysis. EHT accelerating voltage was 20 kV, while the working distance WD was around 9 ± 1 mm. Non-sputtered samples were glued to stubs using carbon glue and then observed.

#### 2.2.2. Test Methods for Concrete Beams on a Semi-Technical Scale

In order to test the self-healing capacity of cracks in cementitious materials using tubes filled with PU on an industrial scale, it was decided to carry out an intermediate stage on a semi-technical scale. The aim was to develop a method for installing the tubes and to see if the self-healing method worked for larger elements with coarse aggregate.

Tests conducted on a semi-technical scale were based on tests of mounting carriers to reinforcement in concrete elements. The aim was to arrange the pipes in such a way as to protect them from changing their position and being destroyed during forming or compacting the mixture. The scope of the research included:

- In stage 1, testing the installation of the tubes directly on the reinforcement was performed; for this purpose, one beam with tubes S3 and S4 was made. These beams were bent 7 days after casting.
- In stage 2, testing the installation of the tubes on the steel mesh and checking the ability of the PU to flow out of the carriers was performed; for this purpose, two beams with S3 and S4 tubes were made. These beams were bent 28 days after casting.
- The method of installing the tubes evolved during the tests. Initially, in stage 1, the tubes were attached to the rods using thin wires, but this did not give the expected result, and the tubes moved when the mould was moved or during vibration, so this method of assembly was abandoned. Finally, in stage 2, the tubes were attached to a square steel mesh (25 × 25 mm^2^) made of wire with a diameter of 0.7 mm. Then the mesh with tubes was attached to steel rods, which ensured the stability of the system.
- In addition, the ability of PU to pour into a crack and fill it was checked. For this purpose, the beams were cracked in a 3-point bending test, and then macroscopic observations were made at the fractures.

#### 2.2.3. Test Methods for Railway Sleepers on an Industrial Scale

After 28 days of casting the sleepers, their mechanical properties were tested. The scope of mechanical tests on prestressed concrete sleepers included:

- Reference sleeper 1 (without tubes) and sleeper 2 with the addition of tubes—the crack resistance of the rail part was tested statically.
- Reference sleeper 3 (without tubes) and sleeper 4 with the addition of tubes—the crack resistance of the rail part was tested dynamically.
- Reference sleeper 5 (without tubes) and sleeper 6 with the addition of tubes—the crack resistance of the central part in the normal position was tested statically.

The tests of the mechanical properties of the sleepers were carried out on a test stand equipped with the Instron Schenck Testing System. The load was carried out with a PL 1000 kN cylinder.

##### The Crack Resistance of the Rail Part Tested Statically

**Figure 10:**
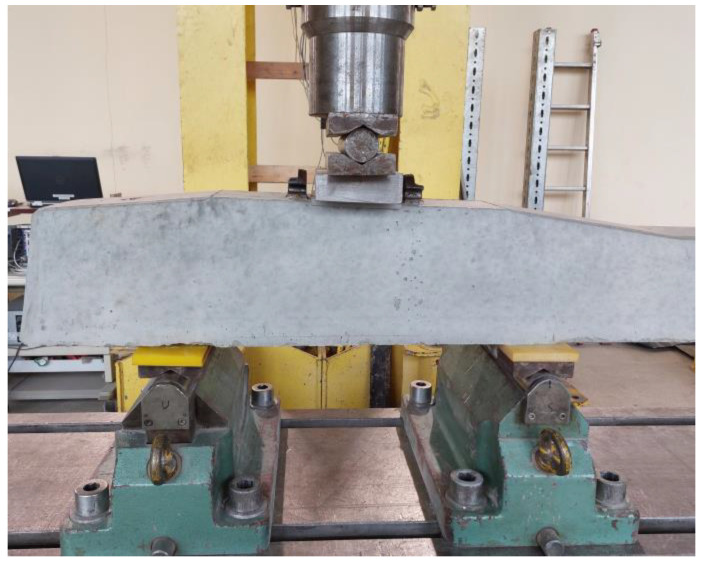
Photo of loading the rail part in the normal position.

Sleepers 1 and 2 were tested in the rail area under static load (Figure 10). The course of the test was in accordance with the standard [1], with the exception of an additional stage for qualitative assessment of crack healing (PU leakage from the underside of the sleeper) during the loading process.

Initially, a load of 161.20 kN was applied to the sleepers, and then it was gradually increased by 10 kN until the first crack appeared. After observing the first crack, the sleeper was unloaded, and then a load of 10 kN greater than the previous value was applied. This process was repeated until a crack width of 0.05 mm was reached after the load was removed. Then the load was continued, gradually increasing it by 10 kN until a crack of 0.5 mm appeared. After reaching this state, the reference sleeper was destroyed. In this way, the value of the Fr_B_ force was determined. On the other hand, for the sleeper modified with PU-filled tubes, after reaching the crack width of 0.5 mm, the loading process was temporarily interrupted in order to qualitatively identify the self-healing process (inspection of whether there is an outflow of the healing agent observed from the underside of the sleeper). Then the sleeper was destroyed.

##### The Crack Resistance of the Rail Part Tested Dynamically

Sleepers 3 and 4 were tested for crack resistance of the rail part (Figure 10) under dynamic load using the standard procedure [1]. The frequency of 4 Hz was assumed for the study. The initial load was in the range of 50–161.10 kN. The upper limit of the load was gradually increased at various stages of the test, going through the crack value after unloading of 0.05 mm up to the value of 0.5 mm. Then, the maximum value of the loading force was reached, and the sleeper, both the reference and modified with PU-filled tubes, was destroyed.

##### The Crack Resistance of the Central Part Tested Statically

**Figure 11:**
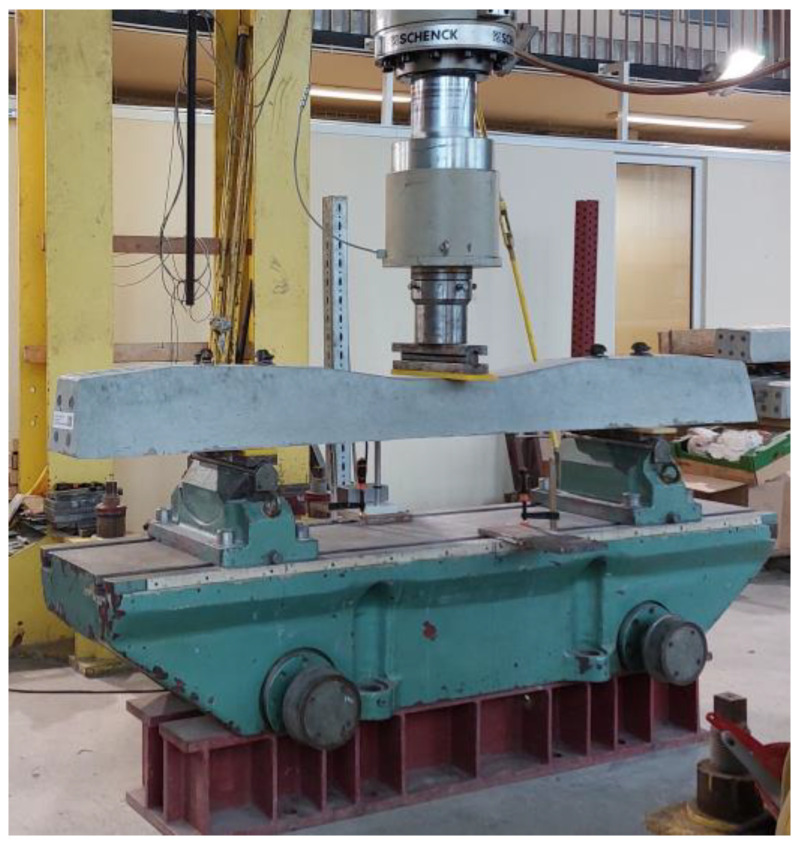
Photo of loading the central part in the normal position.

Sleepers 5 and 6 were tested for crack resistance in the central part. The central part was subjected to the load in the normal position (Figure 11) by applying the load statically in accordance with the standard procedure [1].

In the first stage of the test, the sleeper was loaded with a force of 27.61 kN. Then, the load was gradually increased by 5 kN, observing at what force value (Fc_r_) the first crack appeared, and then until a crack with a width of 0.5 mm appeared. In the case of the reference sleeper, the load was continued until the maximum force Fc_B_ was reached. However, in the case of the sleeper modified with PU-filled tubes, after reaching a crack with a width of 0.5 mm, as in the tests of the rail part, the loading process was stopped to qualitatively identify PU leakage from the underside of the sleeper. Then the load was continued until the sleeper was destroyed.

## 3. Results

### 3.1. Results of Mortar Tests on a Laboratory Scale

#### 3.1.1. Water Permeability Test

**Figure 12:**
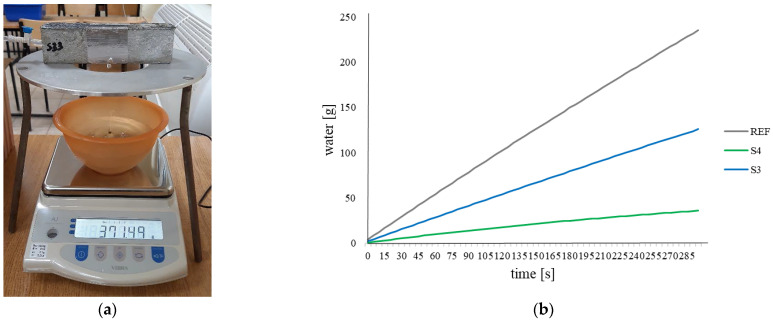
(**a**) Water flow test stand and (**b**) graph of water flow over time for reference samples and samples modified with tubes.

A water permeability test was performed on the cement samples (Figure 12). The graph below shows the water flow versus time for reference samples and samples with tubes (Figure 12).

The average water flow for the reference samples was 47.09 g min^−1^, for the samples with S3 tubes, it was 25.21 g min^−1^, and for the samples with S4 tubes, it was 7.23 g min^−1^. Beams made with the addition of S4 tubes showed the lowest water permeability (over 3 times lower than samples with S3 tubes and over 6 times lower than in the case of reference beams). This proves the highest effectiveness of the healing process for samples with S4 tubes. The level of water flow decreased as more healing agent were applied. S4 tubes were carrier with almost twice the volume of S3 tubes, which can be seen in the test results. Insufficient amounts of the applied healing agent may cause only partial filling of the crack and thus minimize the effects of the self-healing process.

Based on the obtained results of water flow in the reference and modified beams, the self-healing efficiency of the samples was calculated. The sealing efficiency for samples modified with S3 tubes was 46%, while for beams with S4 tubes, it was 85%. By using carriers with a larger cross-sectional diameter and, thus, a larger volume of the healing agent, a greater reduction in water permeability was obtained.

#### 3.1.2. Water Absorption Test

**Figure 13:**
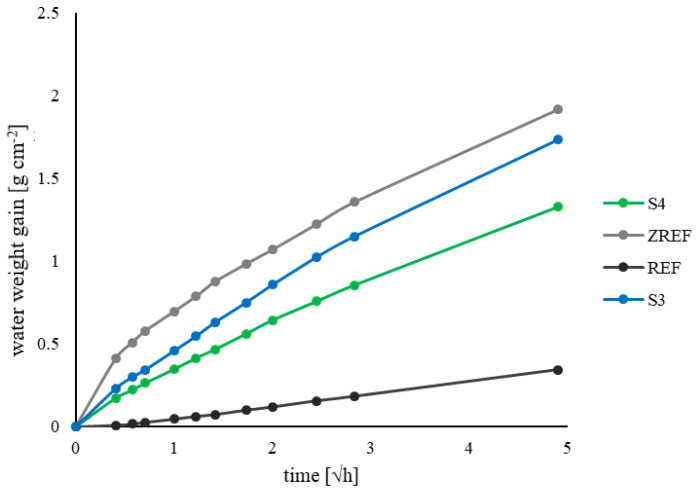
Graph of water absorption (water weight gain over time) for reference samples and samples modified with tubes.

The results of the water absorption test are presented in the graph of the time root vs. water weight increase (Figure 13).

The obtained results indicate the limitations of the absorption for samples modified with tubes filled with the healing agent in relation to cracked reference samples (ZREF). Better results were obtained for samples with the addition of S4 tubes. For reference samples REF, the maximum weight gain was 0.34 g cm^−2^ after 24 h of testing; for samples modified with S4 tubes, it was 1.33 g cm^−2^; for samples S3, this value was 1.73 g cm^−2^; and for cracked reference samples, it was 1.92 g cm^−2^.

The sealing efficiency coefficient (SE) was calculated based on Formula (2) and obtained at a level of 26% for S3 samples and 48% for S4 samples. Due to the larger volume of the healing agent in the S4 carrier, the healing efficiency associated with filling the crack with polyurethane was clearly higher compared to the samples with the S3 carrier.

#### 3.1.3. Macroscopic Observations to Assess the Ability of the Healing Agent to Penetrate the Crack

**Figure 14:**
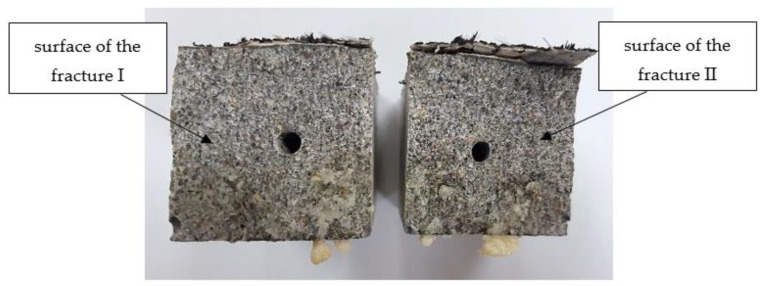
Photo of the cross-section of the sample after the healing process.

In order to quantify the coverage of the beam fracture surface by the released PU, after the water permeability test, the samples were completely broken (Figure 14).

**Figure 15:**
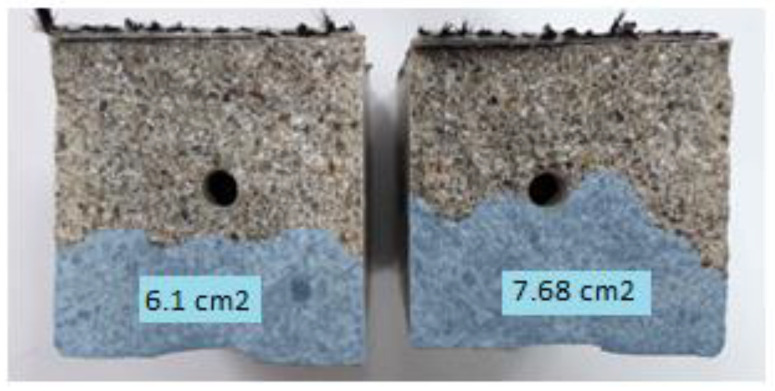
Photos of sample fractures after the treatment process.

It was observed that the surfaces of the fractures were covered with a layer of polymer, which indicates that it flowed away and filled the original crack. Although the beams were always stored with the crack downwards, resin was observed in most of the fractures, not only under but also above the tubes. By outlining the resin covering the fractures of the beam, the surface areas covered with the healing agent were calculated. An exemplary photo of a fracture with marked leak spots is shown in the photo below (Figure 15).

The average coverage of the fracture surface with the healing agent for samples S3 was 5.37 cm^2^, which is about 34% of the area, while for samples S4, it was 6.77 cm^2^, which is more than 42% of the cross-sectional area of the beam. Beams with a higher content of the healing agent, as in previous studies, showed better results.

#### 3.1.4. Microstructure Observations

**Figure 16:**
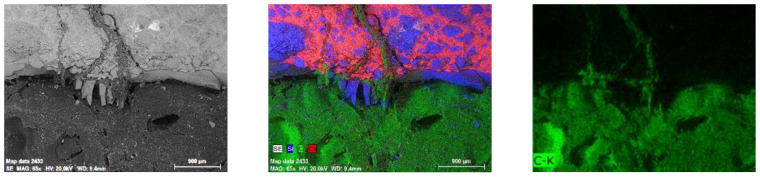

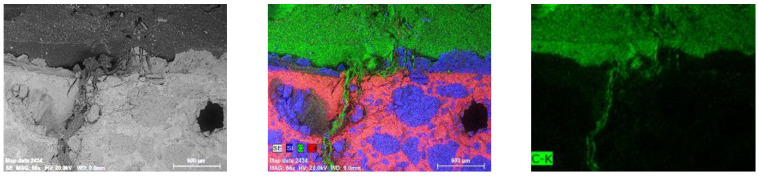
SEM image of a fragment of a PU-filled crack cut along the tube. Next to it, an analysis of the elemental composition (mapping). Magnification 65×. Red is a marker for calcium, green for carbon, and blue for silicon.

The confirmation of the ability of PU to spread in the resulting crack are images from a scanning microscope along with EDS analysis (Figure 16).

The images and analyses show that the crack is filled along the entire length below the tube and partly above it. Since the sample was not rotated at any stage of the research, this indicates the possibility of PU spreading in the crack, which is very important in terms of closing the cracks in order to extend the durability of the element.

### 3.2. Test Results Obtained on a Semi-Technical Scale

**Figure 17:**
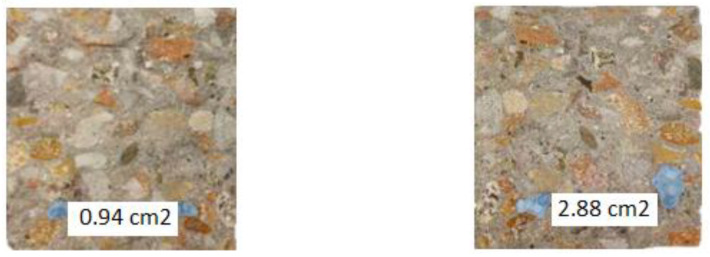
Beam cross-section with S3 tubes.

**Figure 18:**
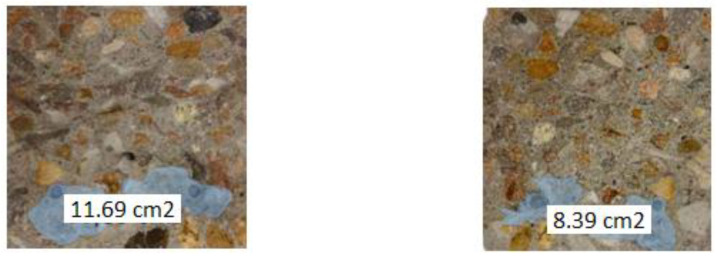
Beam cross-section with S4 tubes.

On a semi-technical scale, after 28 days of curing, the beams with the selected installation method were cracked in a controlled way during a 3-point bending test. Then, they were subjected to another three-point bending test, this time leading to the destruction of the element. Subsequently, the fractures were subjected to macroscopic examination. The photos below show the worst surface coverage for S3 tubes and the best for S4 tubes (Figure 17 and Figure 18).

It was found that in all beams, the S3 and S4 tubes filled with PU were not damaged during concreting. In addition, it was noticed that the carriers remained in their places. The forming process, including vibration, did not change their position. It was also found that the components of the mixture were evenly distributed in the entire beam cross-section. Visual evaluation confirmed the self-healing ability of concrete made in the form of beams with the addition of tubes filled with polyurethane. However, better results were obtained for beams modified with S4 tubes. In this case, a larger surface covered with the healing agent was observed, as well as a better covering of the reinforcement by PU. The fractures and visual inspection of the surface influenced the decision to use S4 tubes on an industrial scale.

### 3.3. Results of Testing Railway Sleepers on an Industrial Scale

#### 3.3.1. The Crack Resistance of the Rail Part Tested Statically

**Figure 19:**
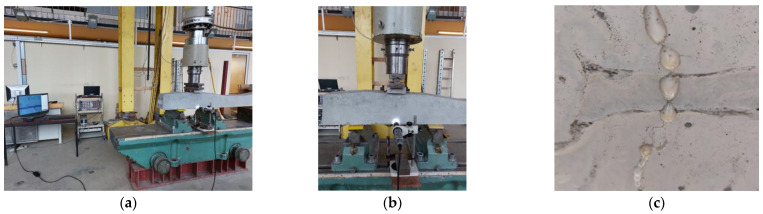
Static testing of the rail part: (**a**) test stand for crack resistance testing; (**b**) a crack on the side surface of the sleeper, created during the test; and (**c**) PU leakage visible from the underside of the sleeper.

**Figure 20:**
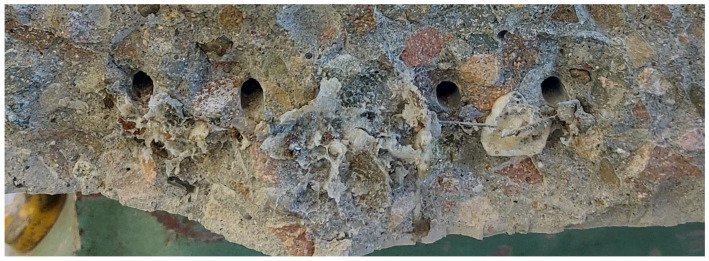

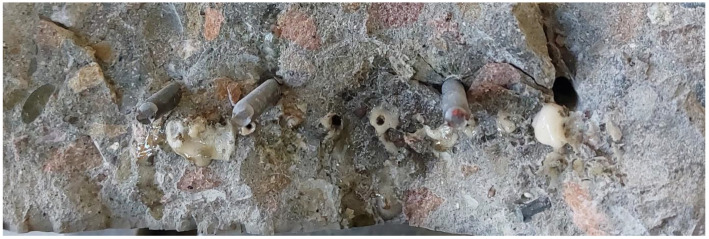
Cross-section of the sleeper in the rail part with visible poured PU.

The reference and modified sleepers were tested in the rail part under static load. The several stages of the study are shown in Figure 19 and Figure 20. The obtained results are presented in Table 2 in relation to the requirements of the national railway infrastructure manager (PKP PLK) [29].

Very similar force values were obtained at the same test stages for both railway sleepers. It can be concluded that the addition of PU-filled tubes did not negatively affect the mechanical properties.

All the tubes survived the process of filling and compacting the concrete mix in the moulds. They also did not change their position during casting. The polyurethane contained in the carriers was released during cracking. It flowed out of the tubes and filled the crack, both below and above the carrier. Some of the PU was locally visible outside the sleeper (leaked outside) (Figure 19). Upon breaking the sleepers, both the bonded polyurethane and the fresh polyurethane still flowing out of the glass tubes could be observed.

#### 3.3.2. The Crack Resistance of the Rail Part Tested Dynamically

**Figure 21:**
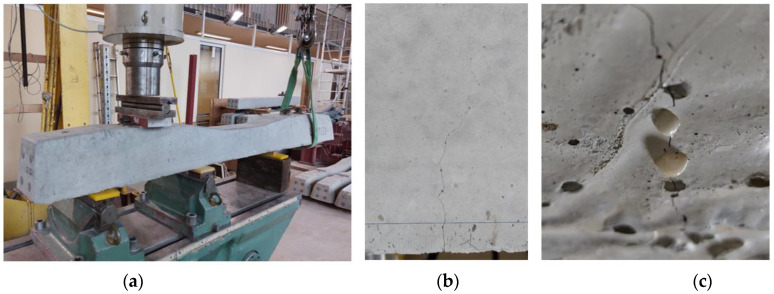
Dynamic testing of the rail part: (**a**) test stand, (**b**) a crack on the side surface of the sleeper, created during the test, and (**c**) PU leakage visible from the underside of the sleeper.

**Figure 22:**
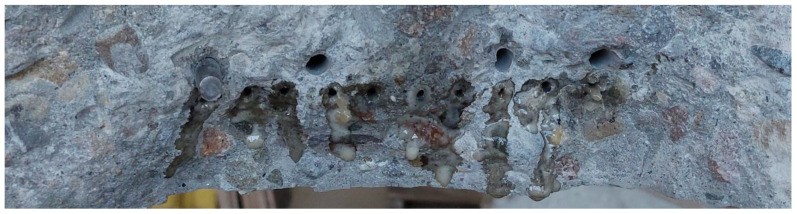
Cross-section of the sleeper in the rail part after the dynamic test. Visible PU leakage from the tubes.

The crack resistance test of the rail part under dynamic load was carried out for the reference sleeper and for the sleeper modified with tubes. As in the case of static studies, the effects of the self-healing process were documented in photographs (Figure 21 and Figure 22). The obtained results were compared with the WTWiO [29] requirements (Table 3).

Again, as was in the case with sleeper 2, all the tubes survived the concreting process here as well. During the test, the carriers ruptured, releasing the healing agent. Observing the fracture, it can be seen that some of the PU was foamed, and some was still leaking from the tubes.

#### 3.3.3. The Crack Resistance of the Central Part Tested Statically

**Figure 23:**
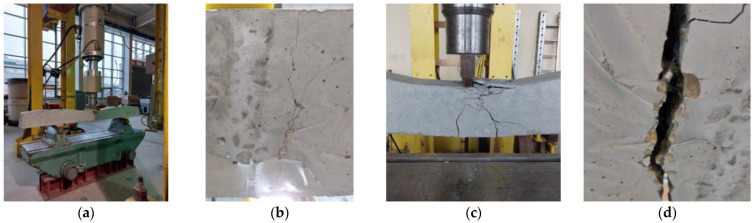
Static test of the middle part in the normal position: (**a**) prestressed concrete sleeper laid for testing the middle part in the normal position, (**b**) a crack with leaking PU visible from the underside of the sleeper, (**c**) destruction of the sleeper, and (**d**) a crack in the process of destruction.

The crack resistance test in the central part was also carried out for the reference sleeper, and the sleeper was modified with PU-filled tubes. The obtained results and the course of the study are presented in Figure 23 and Table 4.

In the case of the sleeper loaded in the central part, the destruction occurs in several planes, including the crushing of the concrete in the upper (compressed) part. In the planes intersecting the tubes, leakage and polymerization of the healing agent were identified.

## 4. Discussion

The obtained test results confirm the possibility of producing pre-stressed concrete railway sleepers modified with PU tubes to increase their durability. They also show that the PU released from the tube fills the crack. This is very important because cracking the sleeper disqualifies it as it does not meet the durability requirements, but cracked sleepers still meet load-bearing requirements and carry loads. Filling the crack with PU released from the tube will allow the railway sleeper to continue to be used despite its cracking. In this aspect, it is also important to test the durability of the PU filling the crack by determining its resistance, primarily to multiple dynamic deformations caused by passing rolling stock. Such tests should be carried out on elements on a real (industrial) scale. Due to the size of the test elements and the impossibility of cutting out a part of the sleeper (because it is a prestressed element), durability tests are difficult. These studies require the implementation of another research program, including, in addition to visual assessment, the use of another method for assessing the effectiveness of self-healing in railway sleepers. It seems that one of the tests for the tightness of a filled crack is to determine the capillary rise of water in the crack created or to perform a water permeability test (similar to the laboratory method used in the case of mortars).

So far, there are no reports in the world literature about the use of such a solution in railway practice. Therefore, it seems reasonable to construct a railway surface using modified self-healing pre-stressed concrete railway sleepers.

## 5. Conclusions

Based on the conducted research and analysis, it was shown that it is possible to achieve the potential of autonomous self-healing of cementitious materials through the application of tubes filled with a healing agent, thus increasing the durability of pre-stressed concrete railway sleepers after they are cracked.

In all tested elements, the self-healing process was initiated by cracking the matrix, which resulted in the cracking of the carrier (glass tubes) and the release of the healing agent (PU). As it was shown in the research, the applied healing agent filled the initiated crack. In the case of tests conducted on the cement mortar beams, studies have shown that the greater the amount of the healing agent, the greater the effectiveness of self-healing. Insufficient amounts of the applied healing agent may only partially fill the crack and thus minimize the effects of the self-healing process. The surface that was covered with polyurethane for S4 beams is on average 42% of the entire cross-section, and for S3, about 34%. However, the effectiveness of separating the material from the water medium is not affected by the surface covered with polyurethane, but only by the fact that the crack has been filled along its entire length, thereby cutting off the material from the external environment (in this case, water). The sealing efficiency in the water permeability test for samples modified with S3 tubes is 46%, while for beams with S4 carrier, it is 85%. The sealing efficiency factor (SE) for the water absorption test was 26% for the S3 samples and 48% for the S4 samples. Observations of the microstructure carried out under a scanning microscope confirmed the ability of the polymer to migrate in the damaged cement matrix and thus the ability to seal the crack. Moreover, the polyurethane was in crack, usually all the way below the tube and partly above it.

The tests of concrete beams were an intermediate stage between the tests of beams on a laboratory scale and the tests of railway sleepers. They made it possible to develop a method of tube application in larger elements. As a result, it was decided to use tubes with a larger diameter (S4), which were then attached to a steel mesh mounted to the reinforcement, thus ensuring the stability of the system.

The last stage of the work was the production of prefabricated structural elements (railway sleepers) modified with PU-filled tubes. The purpose of the proposed modification was to ensure the durability of the sleepers after they were cracked. It is worth noting that these tests are conducted on real structural elements, which is not common in world literature. The elements were subjected to normalized loads, causing their expected damage in the form of cracks, which led to the release of the healing agent. A thorough inspection of the tested sleepers after the healing process showed that all carriers survived the casting process (concreting, intense vibration, etc.) and did not move. During the tests of the sleepers, after they were cracked, the polyurethane was released, and the crack was filled. No deterioration of the mechanical properties of the sleepers was observed due to the modification with PU tubes. It should be emphasized that the modified sleepers met the requirements of the national railway infrastructure manager (acceptance criteria point 4.4 of the standard set out in the Technical Conditions for the Execution and Acceptance of prestressed concrete sleepers and bearers (WTWIO-ILK3a-5187/01/05 (Id-101)) in terms of checked properties.

## Figures and Tables

**Table 1:** Identification of tested polyurethane samples.

Sample Identification	PU0	PU1	PU2	PU3	PU4	PU5
Water content %	0	1	2	3	4	5
Picture	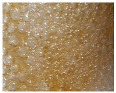	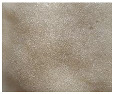	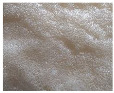	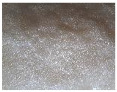	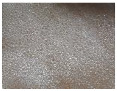	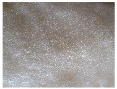

**Table 2:** Summary of the results of the crack resistance tests of the rail part under static load.

	Fr_0_ (kN)	Fr_r_ (kN)	Fr_0.05_ (kN)	Fr_0.5_ (kN)	Fr_B_ (kN)
Guidelines WTWiO/ requirements Id—101	161.20	>200.00	>300.00	---	>450.00
Test results of the reference railway sleeper		211.20	351.20	391.20	688.71
Test results of the railway sleepers with tubes filled with PU		211.20	331.20	381.20	690.86

**Table 3:** Summary of the results of the crack resistance tests of the rail part under dynamic load.

	Fr_0_ (kN)	Fr_r_ (kN)	Fr_0.05_ (kN)	Fr_0.5_ (kN)	Fr_B_ (kN)
Guidelines WTWiO/ requirements Id—101	161.20		>243.00	>356.40	
Test results of the reference railway sleeper		201.20	321.20	421.10	481.20
Test results of the railway sleepers with tubes filled with PU		201.20	291.20	431.20	491.20

**Table 4:** Summary of the results of the crack resistance tests of the central part under static load in the normal position.

	Fc_0_ (kN)	Fc_r_ (kN)	Fc_0.5_ (kN)	Fc_B_ (kN)
Guidelines WTWiO/ requirements Id—101	27.61	>30.00	---	>65.00
Test results of the reference railway sleeper		37.61	62.61	85.61
Test results of the railway sleepers with tubes filled with PU		32.61	52.61	83.86

## Data Availability

Data are contained within the article.
